# Grain size-dependent magnetic and electric properties in nanosized YMnO_3 _multiferroic ceramics

**DOI:** 10.1186/1556-276X-6-201

**Published:** 2011-03-08

**Authors:** Tai-Chun Han, Wei-Lun Hsu, Wei-Da Lee

**Affiliations:** 1 Department of Applied Physics, National University of Kaohsiung, Kaohsiung 811, Taiwan

## Abstract

Magnetic and electric properties are investigated for the nanosized YMnO_3 _samples with different grain sizes (25 nm to 200 nm) synthesized by a modified Pechini method. It shows that magnetic and electric properties are strongly dependent on the grain size. The magnetic characterization indicates that with increasing grain size, the antiferromagnetic (AFM) transition temperature increases from 52 to 74 K. A corresponding shift of the dielectric anomaly is observed, indicating a strong correlation between the electric polarization and the magnetic ordering. Further analysis suggests that the rising of AFM transition temperature with increasing grain size should be from the structural origin, in which the strength of AFM interaction as well as the electrical polarization is dependent on the in-plane lattice parameters. Furthermore, among all samples, the sample with grain size of 95 nm is found to have the smallest leakage current density (< 1 μA/cm^2^).

PACS: 75.50.Tt, 75.50.Ee, 75.85.+t, 77.84.-s

## Introduction

The hexagonal RMnO_3 _(R = rare earth element or Y) compounds present opportunities for the industrial applications due to their unique nature of multiferroism [1]. Namely, the ferromagnetism, ferroelectricity and ferroelasticity occur simultaneously in the same material. The characteristics of multiferroism include a spontaneous magnetization which can be switched by an applied electric field, a spontaneous electrical polarization which can be reoriented by an applied magnetic field, and a strong coupling between these two properties [2]. Owing to the coupling between ferroelectric and magnetic domains, multiferroism is likely to offer a whole range of new applications and phenomena. Specific device applications that have been proposed for these multiferroic materials include the multiple-state memory elements, the transducer with magnetically modulated piezoelectricity, and the electric-field-controlled ferromagnetic resonance devices [2].

Most of hexagonal RMnO_3 _exhibit ferroelectric (FE) transitions at high temperatures (*T*_C _≈ 600 to 1,000 K) and antiferromagnetic (AFM) transitions at low temperatures (*T*_N _≈ 70 to 130 K) with a frustrated triangular arrangement of Mn spins in the hexagonal *c*-plane [1-4]. Additional phase transitions at the temperature below 10 K were observed in the hexagonal RMnO_3 _with the R^3+ ^ion of high magnetic moment, which is related to the R-R exchange correlations [5]. Several attempts have been directed towards the syntheses of new RMnO_3 _compounds and the studies of their related properties [6,7]. In particular, the recent work on the hexagonal RMnO_3 _compounds was focused on the following subjects: (1) the magnetic phases and the magnetic symmetry at low temperatures [8,9], (2) the coupling between the magnetic and FE orderings [10,11], and (3) the strong spin-lattice interaction of the geometrically frustrated Mn-spin system [12]. The studies on YMnO_3_, HoMnO_3 _and LuMnO_3 _indicated that the values of ordering temperatures are associated with the size of R^3+ ^ion. In addition, the size effects in yttrium-based manganites were also reported [13,14]. However, the size effects on the multiferroism remain unclear, and its understanding requires more experimental evidences. In this paper, we prepare a series of YMnO_3 _samples with different grain sizes by a modified Pechini method to study systematically the effect of grain size on their magnetic and electric properties.

## Experimental procedure

The nanosized samples of YMnO_3 _were synthesized by a modified Pechini method using nitrates as metal precursors. First, yttrium nitrate [Y(NO_3_)_3_·6H_2_O] and manganese nitrate [Mn(NO_3_)_2_·4H_2_O] in stoichiometric proportions (1:1 molar ratio) were dissolved in distilled water. Citric acid (C_6_H_8_O_7_) in 1:1 molar ratio with respect to the metal nitrates was added to the solution as a complexant, and the solution was adjusted to a PH value of 6.5 to 7 by adding ammonia. The mixture was dried at 120°C to form a gel, and then the obtained gel was burned until the combustion process was completed. After that, the precursory powders were reground and pressed into the pellets. Finally, the pellets were sintered at different temperatures ranging from 800°C to 1,050°C for 2 h, respectively. Electrodes were applied to both surfaces to measure electrical properties with silver paste.

The crystalline structure and the phase purity of the samples were examined with a typical X-ray diffraction (XRD), acquired by a Bruker D8 Advance X-ray diffractometer (Bruker UK Ltd., Coventry, Warwickshire, UK) equipped with a monochromatized Cu *K*_α1 _radiation and field emission scanning electron microscopy. The magnetization was measured with a Quantum Design superconducting quantum interference device (Quantum Design, Inc., San Diego, CA, USA) with an applied magnetic field of 500 Oe. For the dielectric measurements, a capacitance bridge (Agilent 4284A; Agilent Technologies, Inc., Palo Alto, CA, USA) hooked to a probe station with a closed-cycle low temperature system was used. The leakage currents of the samples were measured using a commercial FE test system (TF Analyzer, aixACCT Systems GmbH, Aachen, Germany).

## Results and discussion

**Figure 1:**
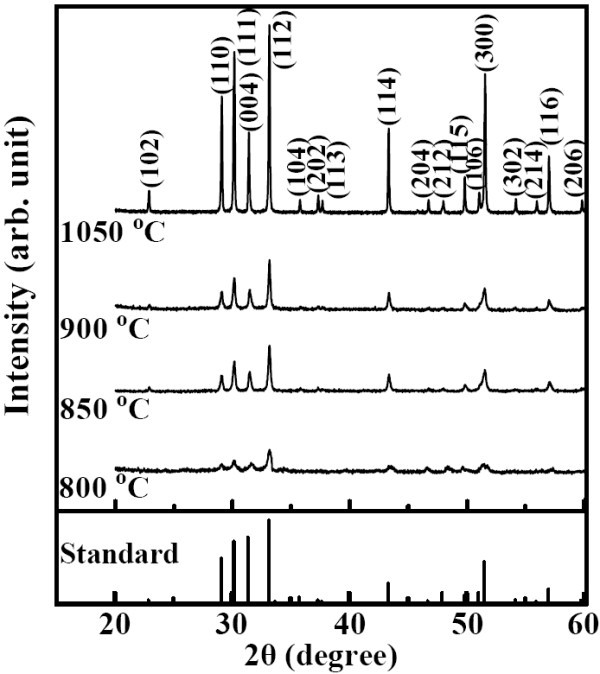
**The standard reference and the XRD patterns of YMnO_3 _samples sintered at different temperatures**. Samples were sintered at temperatures ranging from 800°C to 1,050°C for 2 h, respectively.

**Figure 2:**
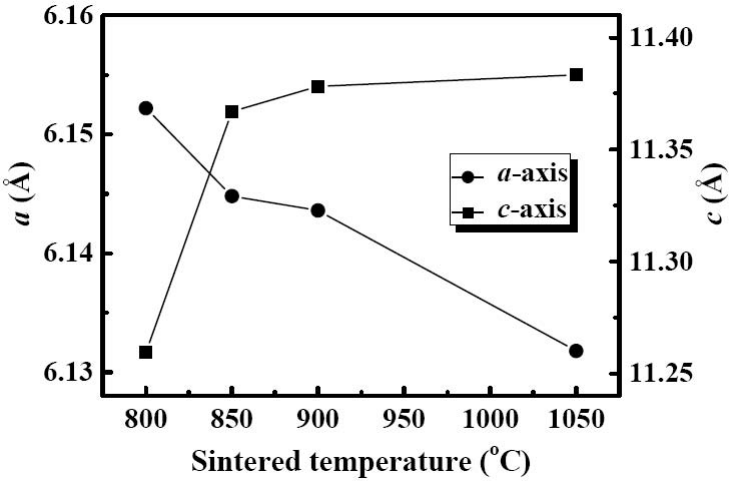
**The evolution of lattice parameters for YMnO_3 _samples sintered at different temperatures**. The uncertainty is contained within the area of the suitable mark.

**Figure 3:**
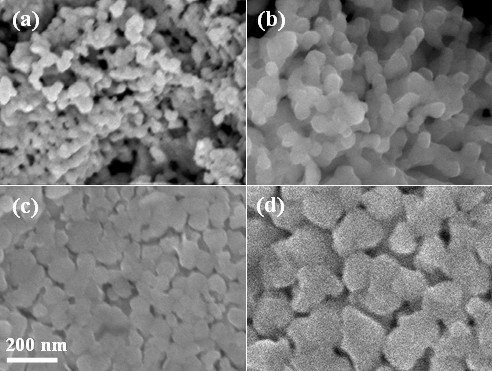
**The SEM micrographs for YMnO_3 _samples**. Samples sintered at **(a) **800°C, **(b) **850°C, **(c) **900°C, and **(d) **1,050°C, respectively.

Figure 1 shows the XRD patterns of the YMnO_3 _samples sintered at different temperatures from 800°C to 1,050°C. Based on the standard reference, all the observed peaks can be indexed on the basis of a hexagonal unit cell of space group P6_3_cm (JCPDS:25-1079), suggesting that all samples are pure phases without any impurity. In addition, with the increase in sintering temperature, there is a gradual intensity increasing and narrowing of the diffraction peaks, indicative of better crystallization and the grain growth. The lattice parameters were determined by Rietveld refinement method and shown in Figure 2. With increasing of sintering temperature, the value of *c *lattice parameter is slightly expanded, while the value of *a *lattice parameter decreased. The typical scanning electron microscopy (SEM) images of the YMnO_3 _samples sintered at different temperatures are shown in Figure 3. From the images, it can be found that the grain size becomes larger as the sintering temperature increases. The estimated average grain size is about 25, 45, 95, and 200 nm for the samples sintered at 800°C, 850°C, 900°C, and 1,050°C, respectively.

**Figure 4:**
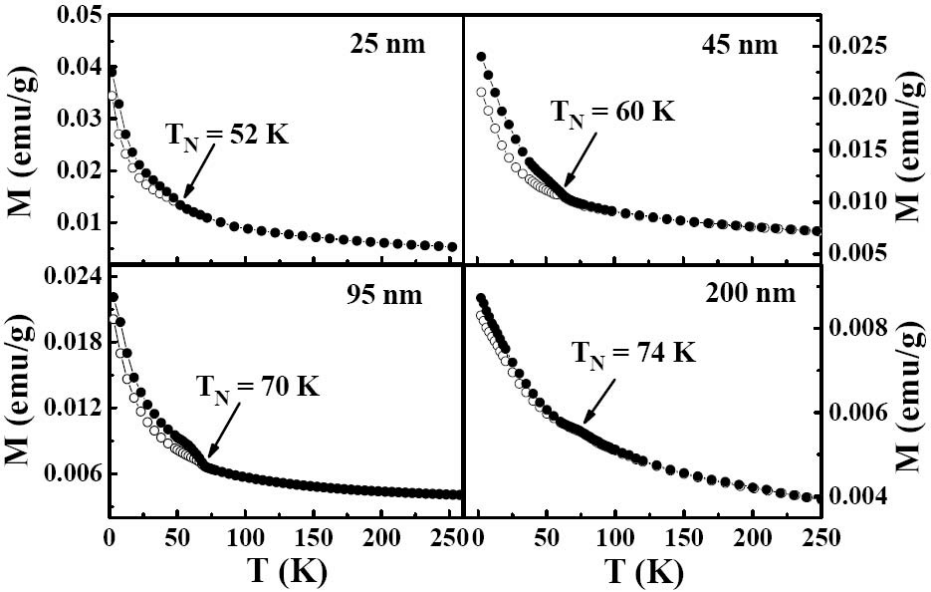
**Temperature dependence of magnetization for the YMnO_3 _samples with different grain sizes**. Open symbols are the data with the ZFC while the solid ones with FC mode.

**Figure 5:**
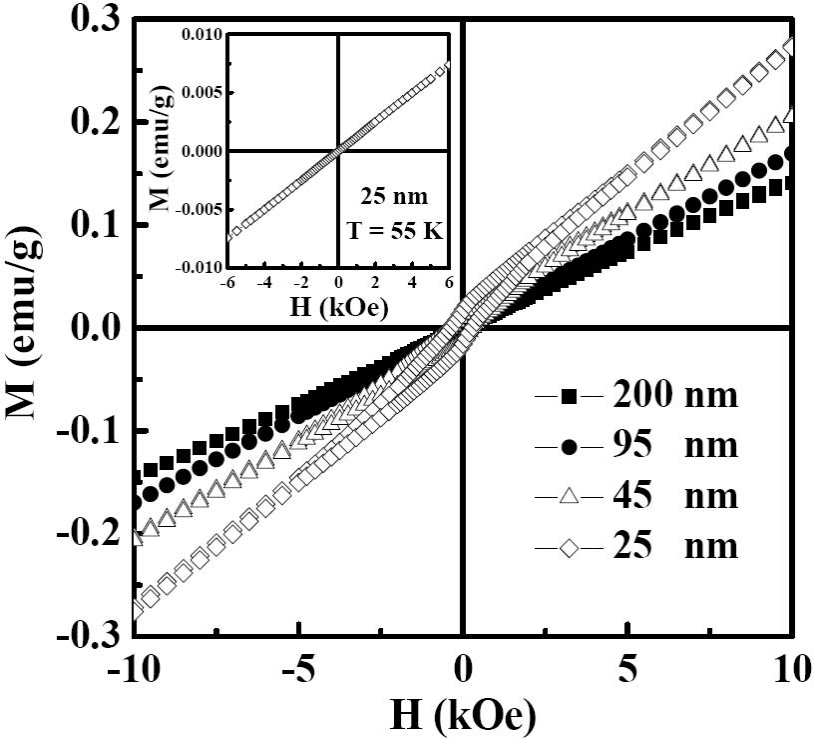
**Magnetic hysteresis loops at 5 K for the YMnO_3 _samples with different grain sizes**. Inset: magnetic hysteresis curve at 55 K for the sample with grain size of 25 nm.

The temperature-dependent magnetization curves *M*(*T*) were measured in a magnetic field of 500 Oe under the conditions of zero-field-cooled (ZFC) and field-cooled (FC). Figure 4 displays the temperature dependence of magnetization for the powders with different grain sizes. Open symbols are the data with the ZFC mode, while the solid ones with FC mode. As can be seen, typical AFM to paramagnetic (PM) phase transition is observed for the sample with grain size of 200 nm, and the Néel temperature (*T*_N_) is about 74 K. As the grain size decreases, the value of *T*_N _shifts to the lower temperatures and is equal to 52 K for the sample with grain size of 25 nm. This size-dependent *T*_N _is similar to the observation in the BiFeO_3 _nanoparticles [15], where the increase in *T*_N _with increasing size has been discussed both in terms of phenomenological scaling relations and possible correlations with the decreasing electrical polarization. To further explore the magnetic properties of the samples, magnetic hysteresis loops for the YMnO_3 _samples with different grain sizes have been measured at 5 K, as presented in Figure 5. For the samples with grain size of 25 and 45 nm, weak ferromagnetic (FM) behavior is observed with corresponding coercivity (Hc) about 395 and 260 Oe, respectively. The inset in Figure 5 shows the magnetic hysteresis curve for the sample with grain size of 25 nm has been measure at 55 K. It indicates the PM behavior which confirms that the FM component disappears above *T*_N_. Therefore, the weak FM component does not come from FM impurity phase. As the grain size increases, the weak FM behavior transforms into paramagnetism. Similar effect of grain size on magnetism was also reported in nanosized YMn_2_O_5 _[16] and BiFeO_3 _particles [17]. In fact, weak surface FM component is a universal feature for nanosized AFM systems, which is attributed to the deviation of the AFM arrangement to the disordered surface spin due to the lattice strain [17,18]. Based on the above consideration, the magnetic structure of the nanosized YMnO_3 _can be considered as a core/shell system, where the inner part of the particle is AFM phase and the surface is FM component.

**Figure 6:**
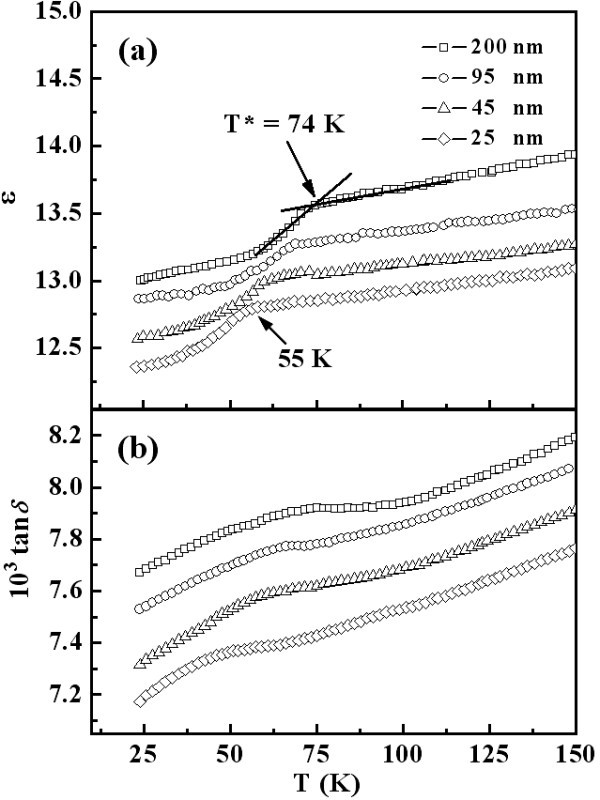
**Temperature-dependent (a) dielectric constant (*ε*), and (b) loss tangent for the YMnO_3 _samples**. Samples have different grain sizes (25 nm to 200 nm).

Figure 6 shows the temperature-dependent dielectric permittivity *ε*(*T*) and loss tangent (tan*δ*) at 100 kHz for all measured YMnO_3 _samples. In Figure 6a, the dielectric anomalies are observed at T* which is defined as the crossing point of two slopes as indicated by arrows. It shows that the T* shifts from 55 to 74 K with increasing grain sizes from 25 to 200 nm. As clearly apparent in Figure 6b, the positions of the broad peaks for the YMnO_3 _samples with different grains sizes are near their T*. Moreover, the enhanced dielectric response observed for YMnO_3 _with larger grains is similar to previously reported results for BaTiO_3 _dielectrics [19]. The observed systematic shift in the temperatures of magnetic transition and dielectric anomaly demonstrates a strong correlation between magnetic ordering and electric polarization in nanosized hexagonal YMnO_3 _ceramics. As to the coupling between antiferromagnetism and dielectric property, Katsufuji et al. [20] suggested that the dielectric anomaly was caused by the magnetic-ordering-dependent electronic excitation gap *E*_g _in *ab*-plane. According to this model, the change of AFM ordering pattern can induce dielectric anomaly via the change of *E*_g_, in a formula of *ε *= 1/*E*_g_^2^. Therefore, one can understand that the shift in the temperature of dielectric anomalies is related to the AFM interaction through the variation of Mn-O bond length with change the lattice parameters. In addition, the systematical change in the lattice constant *a *plays an important role since the strength of AFM interactions strongly depends on the bond length of Mn-O. In general, the strength of AFM interaction can be written as [21]:(1)

where the sum is over the nearest neighbors and  is a spin operator. The parameter *J *is proportional to the inverse of the distance between two nearest spins. Therefore, the reduction in *a*-parameter leads to the enhancement of *J *and hence to the rising of AFM transition temperature.

**Figure 7:**
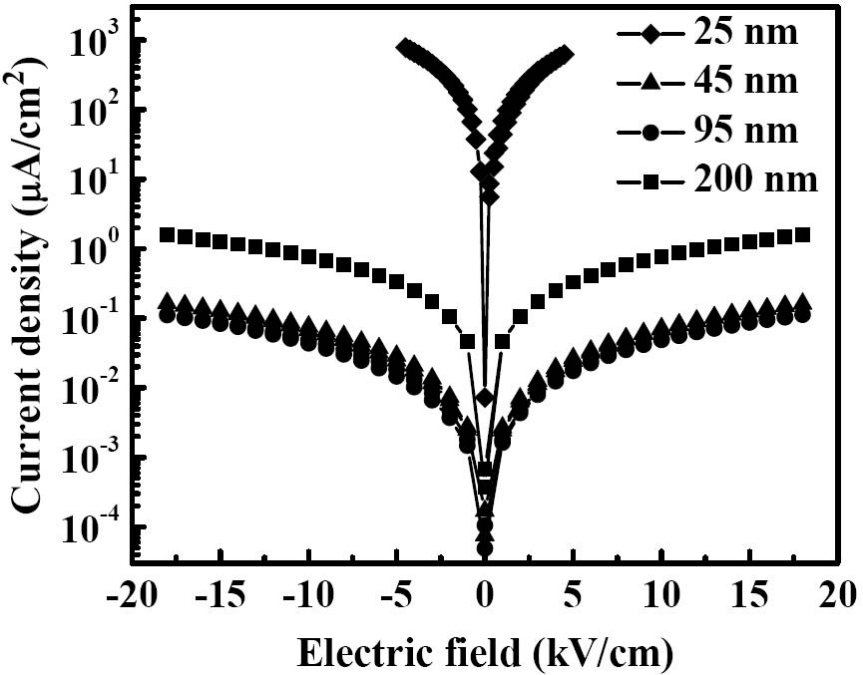
**Leakage current as function of applied electric field for the YMnO_3 _samples**. Samples have different grain sizes (25 nm to 200 nm).

To further probe the electrical leakage effect, the leakage current were measured for all the samples at room temperature as shown in Figure 7. The leakage current density is large (> 100 μA/cm^2^) for the sample with grain size of 25 nm. On the other hand, the leakage currents are much decreased by about four orders of magnitude for the samples with grain size larger than 45 nm. In addition, it is not expected that the sample with larger grain size of 200 nm is not the less leaky sample. As for the improvement of the leakage properties, it should be associated with the high denseness of the ceramics [22].

## Conclusions

In summary, a series of hexagonal YMnO_3 _samples with different grain sizes are synthesized by a modified Pechini method. The magnetic susceptibility indicates that with increasing grain size from 25 to 200 nm, the AFM transition temperature increases from 52 to 74 K. At the same time, a corresponding shift of the dielectric anomalies is observed, which suggests a strong correlation between the magnetic ordering and the electric polarization. Since the electronic excitation gap is inversely proportional to the dielectric permittivity and the spin structure influences the electronic excitation gap, we propose that the coherent shift in the magnetic ordering and the dielectric anomalies to high temperature with increasing grain size is related to the suppression of the in-plane lattice parameter.

## Competing interests

The authors declare that they have no competing interests.

## Authors' contributions

The work presented here was carried out in collaboration between all authors. TH defined the research theme and designed methods and experiments, carried out the laboratory experiments, analyzed the data, interpreted the results and wrote the paper. WH and WL prepared the samples, helped to carry out the laboratory experiments and discussed analyses. All authors read and approved the final manuscript.
